# Abnormal degree centrality and functional connectivity in Down syndrome: A resting-state fMRI study

**DOI:** 10.1016/j.ijchp.2022.100341

**Published:** 2022-10-06

**Authors:** Cristina Cañete-Massé, Maria Carbó-Carreté, Maribel Peró-Cebollero, Shi-Xian Cui, Chao-Gan Yan, Joan Guàrdia-Olmos

**Affiliations:** a Department of Social Psychology and Quantitative Psychology, Faculty of Psychology, Universitat de Barcelona, Barcelona, Spain; b UB Institute of Complex Systems, Universitat de Barcelona, Barcelona, Spain; c Serra Hunter Fellow, Department of Cognition, Development and Educational Psychology, Faculty of Psychology, Universitat de Barcelona, Barcelona, Spain; d Institute of Neuroscience, Universitat de Barcelona, Barcelona, Spain; e CAS Key Laboratory of Behavioral Science, Institute of Psychology, Beijing, China; f International Big-Data Center for Depression Research, Chinese Academy of Sciences, Beijing, China; g Magnetic Resonance Imaging Research Center, Institute of Psychology, Chinese Academy of Sciences, Beijing, China; h Sino-Danish College, Sino-Danish Center for Education and Research, University of Chinese Academy of Sciences, Beijing, China

**Keywords:** Down syndrome, Degree centrality, Seed-based functional connectivity, Brain networks, Functional connectivity

## Abstract

**Background/Objective:**

Neuroimaging studies have shown brain abnormalities in Down syndrome (DS) but have not clarified the underlying mechanisms of dysfunction. Here, we investigated the degree centrality (DC) abnormalities found in the DS group compared with the control group, and we conducted seed-based functional connectivity (FC) with the significant clusters found in DC. Moreover, we used the significant clusters of DC and the seed-based FC to elucidate differences between brain networks in DS compared with controls.

**Method:**

The sample comprised 18 persons with DS (*M* = 28.67, SD = 4.18) and 18 controls (*M* = 28.56, SD = 4.26). Both samples underwent resting-state functional magnetic resonance imaging.

**Results:**

DC analysis showed increased DC in the DS in temporal and right frontal lobe, as well as in the left caudate and rectus and decreased DC in the DS in regions of the left frontal lobe. Regarding seed-based FC, DS showed increased and decreased FC. Significant differences were also found between networks using Yeo parcellations, showing both hyperconnectivity and hypoconnectivity between and within networks.

**Conclusions:**

DC, seed-based FC and brain networks seem altered in DS, finding hypo- and hyperconnectivity depending on the areas. Network analysis revealed between- and within-network differences, and these abnormalities shown in DS could be related to the characteristics of the population.

## Introduction

Down syndrome is the most common genetic condition associated with intellectual disability (ID; Bull, 2020). With medical advancements, life expectancy has greatly increased and has led to the appearance of Alzheimer's disease (AD; Fortea et al., 2020). Recent studies regarding structural and functional magnetic resonance imaging (*f*MRI) have started to emerge, but neuropathology is still unknown, and neuroimaging could be a useful technique to understand brain abnormalities in DS (Baburamani et al., 2019).

Structural studies have demonstrated reduced volume in some regions of the brain (Guidi et al., 2011; McCann et al., 2021). Regarding *f*MRI studies, research using whole brain has highlighted disrupted network connectivity in this population (Anderson et al., 2013; Vega et al., 2015), finding local functional connectivity (FC) differences compared with their peers without ID.

Recent investigations have studied FC in DS using a seed-based approach. In this sense, the default mode network (DMN) has been studied in populations with DS, and a global disruption of FC has been found (DiProspero et al., 2022; Koenig et al., 2021; Rosas et al., 2021; Wilson et al., 2019). Nevertheless, the scarcity of studies in this field hinders the possibility of formulating a prior hypothesis, and therefore, an alternative method that does not assume a prior hypothesis could be more adequate in this population, as the recent study of Csumitta et al. (2022) which studies whole-brain FC and network selectivity in youth with DS.

Degree centrality (DC) is a graph metric that assesses the importance of each node in a brain network, evaluating the connectivity strength to every voxel (Telesford et al., 2011; Zuo et al., 2012). DC offers the opportunity for an unbiased general search of abnormalities within the entire connectivity matrix of the full-brain functional connectome (Zhou et al., 2014), and study functional brain abnormalities at the whole-brain level without prior hypotheses.

FC seems disrupted in DS, but findings until now are not clear, finding in some studies increased FC (Csumitta et al., 2022) and in others reduced FC in DS (Rosas et al., 2021; Wilson et al., 2019). Moreover, the great variability in the age of the participants could hinder the results because of the dementia appearance. Therefore, the aim of this investigation is to (1) perform DC analysis to identify voxels that show altered FC with other voxels, (2) conduct seed-based FC with the areas showing differences between DS and controls to disentangle the underlying mechanism of DS, and (3) perform network analysis using the obtained results of the significant voxels of DC between both populations and the results of the seed-based FC analysis as regions of interest (ROIs).

As main hypothesis in this study, we believe that DC values in whole brain will be different that in controls, as Pujol et al. (2015). The areas that will be settled as seeds (coming from the DC clusters) will also show different FC with other areas of the brain in DS, as this graph metric evaluates the significance of each node in the brain, and therefore it is directly linked with FC (Rubinov & Sporns, 2010; Watts & Strogatz, 1998). Between and within network FC using Yeo et al. (2011) parcellations will be altered in DS involving regions of the cerebellum, frontal lobes and DMN following other findings in this population (DiProspero et al., 2022; Koenig et al., 2021; McCann et al., 2021; Rosas et al., 2021; Wilson et al., 2019). Other authors have found also an anterior-posterior dissociation in the DMN in DS (Rosas et al., 2021) and therefore we expect to find results in the same line.

## Materials and methods

### Participants

Data collection for this project was approved by the Bioethical Committee of the Universitat de Barcelona (03/16/2017). Informed consent was also acquired from all DS and control participants, as well as from the guardians in legal charge of every person with DS.

Twenty persons with DS and twenty non-DS controls matched by chronological age (±2 years maximum in difference of age) and gender were recruited, and the same protocol was applied for both.

For DS participants, recruitment was conducted through different centers attending people with IDs in Catalonia, Spain. The inclusion criteria for this group were as follows: (1) age between 16 and 35 years old and (2) a formal diagnosis of DS. The exclusion criteria for this group were: (1) presence of other comorbid diagnoses implying cognitive dysfunction (2) if the legal guardian's consent could not be obtained, and (3) the person with DS had medication that could affect cognitive function.

Regarding control participants, recruitment was made from the community through advertisements. They had to be matched by gender and age with DS participants and were excluded if they had any psychiatric diagnoses or other disorders affecting cognitive function.

For both groups, if excessive movement was present in the registration of the *f*MRI sequences, the participant was discarded. In the preprocessing section the methods used to exclude a subject for movement will be further explained. IQ was estimated for both groups with the Kaufman Brief Intelligence Test, Second Edition (KBIT-2; Kaufman, 1990). The demographic information of the sample appears in Table 1.

**Table 1:** Participant characteristics.

	DS (mean; SD)	C (mean; SD)	Test (*p value*)
Age (years)	28.67 (4.18)	28.56 (4.26)	*Z* = -0.03 (*p* = .975)
Head motion	0.19 (0.10)	0.08 (0.03)	*Z* = -4.46 (*p* < .001)
Vocabulary KBIT-2 (DS group, n=17)	25.41 (12.23)	71.72 (4.10)	*Z* = -5.06 (*p* < .001)
Matrices KBIT-2 (DS group, n=17)	13.17 (5.44)	39.33 (3.34)	*Z* = -5.06 (*p* < .001)
Total IQ KBIT-2 (DS group, n=17)	43.94 (6.23)	111.05 (7.83)	*Z* = -5.31 (*p* < .001)

DS: down syndrome participants; C: control participants; Z: Z score linked to the Mann–Whitney test; SD: standard deviation

### Measures

Participants were evaluated in two sessions, and the sequence was the same for the DS and control participants.

The data used in the study comprise the following: (1) a usual sociodemographic questionnaire; (2) a checklist for the *f*MRI scanner; and (3) KBIT-2 evaluation. It is important to mention that data were missing for one subject with DS, to whom the KBIT-2 was not administered. Hence, the total sample with KBIT-2 evaluation was *n* = 35 despite the sample for the resting-state *f*MRI measures is 18 persons for each group.

### Imaging acquisition

Brain imaging was performed in a Philips Ingenia 3 MRI scanner T system (Fundació Pasqual Maragall, Barcelona, Spain). All participants underwent a *f*MRI recording sequence: T1, T2, Flair, and resting state. Participants with DS only underwent a total of 6 min (exactly 6.41 mins) in the MRI scanner, whereas control participants underwent a sequence of 10 min. However, to enable group comparison, for this study, only the first 220 volumes of registration were used. Participants with DS underwent a short training to improve their familiarization with the scan and acclimate to the noise and environment. All participants were told to try to stay quiet without movement. Moreover, they should remain awake and with their eyes opened and fixed on a cross symbol on the screen. Participants could choose music to hear during all recordings except in the resting-state scan. None of the subjects included fell asleep during scanning, as self-reported by them after scanning. A T1-weighted turbo field echo (TFE) structural image was obtained for each subject with a 3-dimensional protocol (repetition time (TR) = 2300 ms, echo time (TE) = 2980 ms, 240 slices, and field of view (FOV) = 240 × 240 × 170). The image acquisition was in the sagittal plane. For the functional images, a T2 *-weighted (BOLD) image was obtained (TR = 1750 ms, TE = 30 ms, FOV = 230 × 230 × 160, and voxel size = 3 × 3 × 3 mm, 46 slices). The image acquisition was in the transverse plane.

### Preprocessing

Image preprocessing was performed using the Data Processing Assistant for Resting-State fMRI (DPARSF; Yan & Zang, 2010). Basically, it is based on MATLAB, SPM12 and DPABI (Yan et al., 2016). The preprocessing procedure is described elsewhere (Li et al., 2021).

As DS is a specific population that can present excessive movement, the criterion used to exclude subjects in the sample was that the participants could not exceed the mean of the group plus 2 standard deviations (Yan et al., 2013), estimated with Jenkinson's framewise displacement (FD; Jenkinson et al., 2002). Overall, two persons with DS were excluded, and the final sample was 18 people with DS and 18 controls. Both groups differed in movement as shown in Table 1. The DS group presented a total of 1040 (out of 3780) volumes exceeding 0.2 mm in Jenkinson FD whereas the control participants presented a total of 172 volumes exceeding 0.2 mm in Jenkinson FD. Therefore, scrubbing regression was performed (Yan et al., 2013; Power et al., 2012, 2013) in the final step of the preprocessing in order to control the movement exceeding 0.2 Jenkinson's FD. Moreover, further statistical analyses were performed with the covariate of mean Jenkinson's FD for every subject.

### Degree centrality analysis

Basically, based on the preprocessed data, voxel wise DC was performed using DPABI software. Owing to the uncertainty of interpretation, only positive Pearson correlation coefficients were considered in the DC calculations. As a threshold is usually applied to the typical correlation matrices (van den Heuvel et al., 2008), in this sense, DC was estimated for nodes over a range of thresholds (sparsity range 0.05–0.50). The best threshold was chosen using the area under the curve (AUC). This procedure has been used in previous studies (Yang et al., 2021), and is sensitive at detecting topological alterations of brain disorders (Achard & Bullmore, 2007).

### Seed-based FC

Seed-based FC was estimated using DPABI software. Regions with significant group differences in DC analysis between controls and DS were used as seeds for further resting-state FC. Seed regions were spheres with a radius of 6 mm around the center voxels, and the reference time series for seeds were obtained by averaging the time series of all voxels within the seed region. Correlation analysis was then performed between the seeds and the remaining voxels. Finally, the correlation coefficients were converted into Fisher z values to obtain a z-FC map for further statistical analysis.

### Edge-based functional connectivity

For the network-based analysis, first, the ROIs signals were used to perform the network construction with DPABI. The ROI signals were extracted using the results of the DC significant coordinates (6 coordinates) and the 22 significant coordinates of the seed-based FC. All of them were extracted with a sphere with a radius of 6 mm around the center voxels, and they were used as the nodes to estimate FC.

To better describe significant clusters obtained in network-based contrast, we also classified suprathreshold edges by their membership in the networks defined by Yeo et al. (2011) networks using a script (MATLAB), which identifies the Yeo networks using Buckner and Choi parcellations (Buckner et al., 2011; Choi et al., 2012). The seven networks are the visual network (VN), somatosensory-motor network (SMN), dorsal attention network (DAN), ventral attention network (VAN), limbic network (LN), frontoparietal network (FPN), and DMN.

### Statistical analysis

The data analysis was performed with IBM SPSS (v26) to compare both groups’ characteristics. More concretely, two-group comparisons were tested using nonparametric tests due to the nonapproximation to the normal distribution of the quantitative variables, and *p* < .05 was set as significant.

Statistical analysis regarding DC, seed-based FC and network analysis were performed using DPARSF.

First, to determine if significant differences were found between groups in DC, DPABI was used with a voxel wise two-sample t-test. As mentioned above, both groups differed significantly in head motion; therefore, Jenkinson's FD (Jenkinson et al., 2002) was included as a covariant in all analyses. Significant differences in the study were reported using the criteria of multiple comparisons with the threshold-free cluster enhancement (TFCE), which reaches the best balance between familywise error and test-retest reliability (Chen et al., 2018). A total of 10,000 permutations were performed, and the cluster *p value* was set to *p* < .05.

Second, to determine if significant differences were found in seed-based FC, voxel wise two-sample t tests were performed using the z-FC map, Gaussian random field (GRF) correction of multiple comparisons and Jenkinson's FD as a covariant.

Finally, to perform network-based FC analysis, the network matrix of each subject was used, and a t-test was performed using false discovery rate (FDR) correction (Chen et al., 2018) with nonparametric permutation for the 28 coordinates used. Jenkinson's FD was also set as a covariant.

## Results

### Participant characteristics

In Table 1, the participants’ characteristics are shown. As shown, significant differences were found in head motion and all subtests of KBIT-2.

### Degree centrality

**Figure 1:**
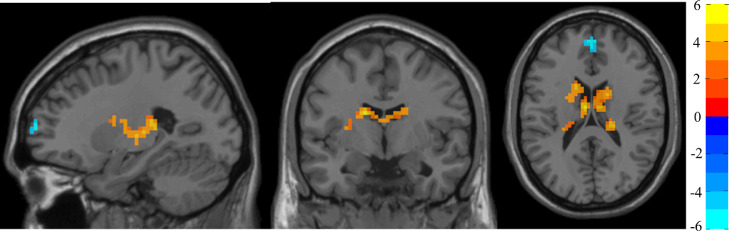
DC analysis. Two-sample t-test results corrected by TFCE are presented. The area in blue represents a significantly decreased DC value in DS patients compared with controls; the area in yellow and red represents a significantly increased DC value in DS patients compared with controls.

Table 2 shows the significant differences between groups in DC with MNI coordinates. Figure 1 shows the graphical representation using DPARSF.

**Table 2:** Significant between-group differences in DC.

Comp.	Area	Nr. Vox	*t(*peak)	Peak MNI coordinates (mm)			AAL peak region
DS>C	Left temporal lobe	10	6.00	−48	−27	−27	Temporal_Inf_L
	Right frontal and temporal lobe	157	5.89	12	24	−21	Rectus_R
	Left caudate	343	5.59	−6	−9	18	Thalamus_L
	Left rectus	2	3.95	0	45	−27	∼Rectus_L
C>DS	Left frontal lobe	21	−5.57	0	54	18	Frontal_Sup_Medial_L
	Left frontal lobe	12	−5.42	−27	66	6	Frontal_Mid_L

DS: down syndrome participants; C: control participants; MNI: Montreal Neurological Institute; ∼: approximately, AAL atlas area closer to the t peak.

Compared with matched controls, people with DS showed significantly increased DC in the temporal and right frontal lobe, as well as in the left caudate and rectus. Controls showed significantly increased DC compared with DS in in regions of the left frontal lobes.

### Seed-based FC analysis

Table 3 shows the significant clusters found in seed-based FC. It is important to highlight that the right rectus and the left thalamus did not present any significant difference between both groups.

**Table 3:** Significant between-group differences in seed-based FC using the significant clusters of DC results as seed regions.

Seed region	Significant differences	T (voxels)	Comp.
Temporal_Inf_L	Cerebellum_Crus_1_R	4.46	DS>C
	Temporal_Sup_L	4.84	DS>C
	Lingual_R	5.62	DS>C
	Precuneus_L	5.77	DS>C
	Precuneus_R	6.63	DS>C
Thalamus_L	Cerebellum_9_R	4.89	DS>C
	Hippocampus_L	6.28	DS>C
	Lingual_R	4.72	DS>C
	Occipital_Mid_L	5.19	DS>C
	Lingual_L	5.16	DS>C
	Frontal_Inf_Oper_L	4.18	DS>C
	Cuneus_L	5.14	DS>C
	apr Postcentral_L	4.22	DS>C
	Precentral_R	4.32	DS>C
	Cingulum_Mid_L	4.38	DS>C
	Supp_Motor_Area_L	5.02	DS>C
Frontal_Sup_Medial_L	Cingulum_Ant_L	−4.86	C>DS
	Precuneus_L	4.97	DS>C
	Angular_L	−5.03	C>DS
	Frontal_Sup_Medial_L	−4.86	C>DS
	Frontal_Mid_R	−5.33	C>DS
Frontal_Mid_L	Precuneus_R	−4.42	C>DS

DS: down syndrome participants; C: control participants

### Network-based analysis

As previously stated, network-based analysis was performed using the 6 significant DC clusters and the 22 significant clusters of the seed-FC analysis.

All regions were classified using the Yeo et al. (2011) parcellations. Table 4 shows the classification.

**Table 4:** Classification of the regions in Yeo networks.

Others	VN	SMN	DAN	VAN	LN	FPN	DMN
Thalamus_L	Lingual_R	Temporal_Sup_L	Precuneus_L	Cingulum_Mid_L	Temporal_Inf_L	Frontal_Mid_R	Frontal_Sup_Medial_L
Precuneus_R	Lingual_R	apr Postcentral_L	Precentral_R		Rectus_R		Frontal_Mid_L
Hippocampus_L	Occipital_Mid_L	Supp_Motor_Area_L			apr Rectus_L		Cerebellum_Crus_1_R
Cingulum_Ant_L	Lingual_L				Cerebellum_9_R		Frontal_Inf_Oper_L
	Cuneus_L						Precuneus_L
							Angular_L
							Frontal_Sup_Medial_L
							Precuneus_R

VN: visual network; SMN: somatosensory motor network; DAN: dorsal attention network; VAN: ventral attention network; LN: limbic network; FPN: frontoparietal network; DMN: default mode network.

**Figure 2:**
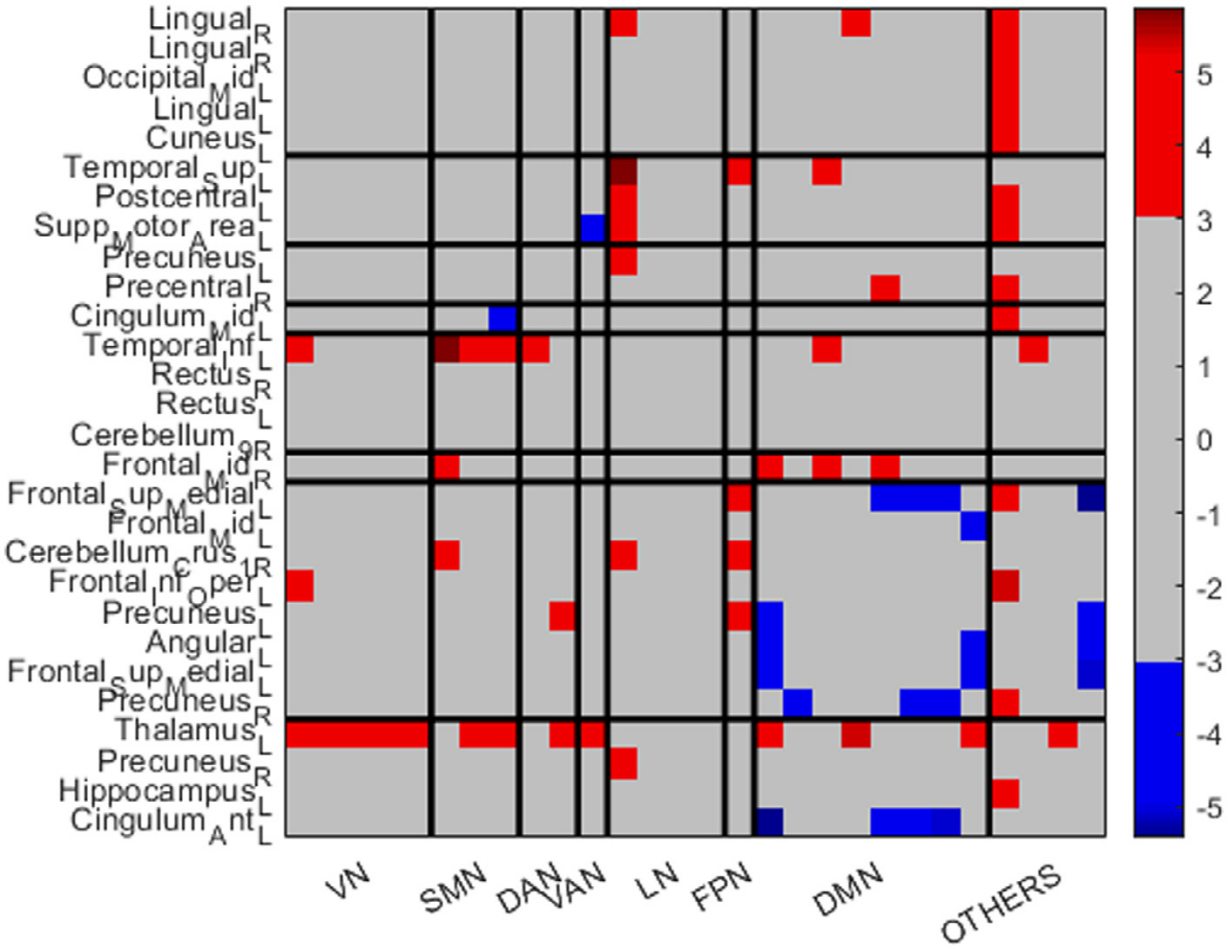
Edge plot matrix. Blue colors show areas that have significantly increased connectivity in control participants compared to DS. Red areas show significantly increased connectivity in DS patients compared with controls.

**Figure 3:**
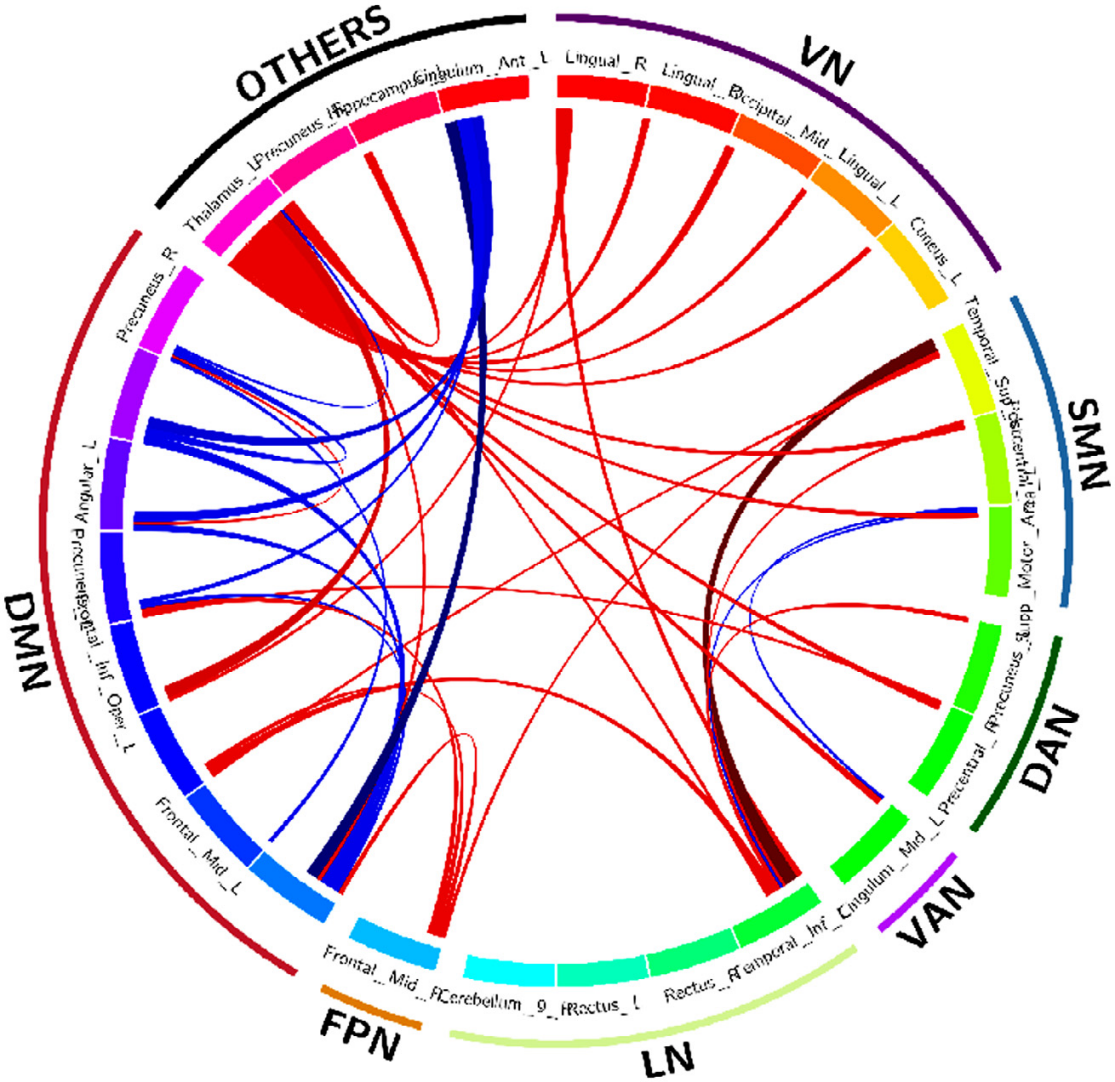
Spatial connectogram of differences in connectivity between DS and controls generated using the Circos tool (Krzywinski, et al., 2009). Blue lines show areas that have significantly increased connectivity in control participants compared to DS. Red lines show areas that have increased connectivity in DS patients compared with controls.

**Figure 4:**
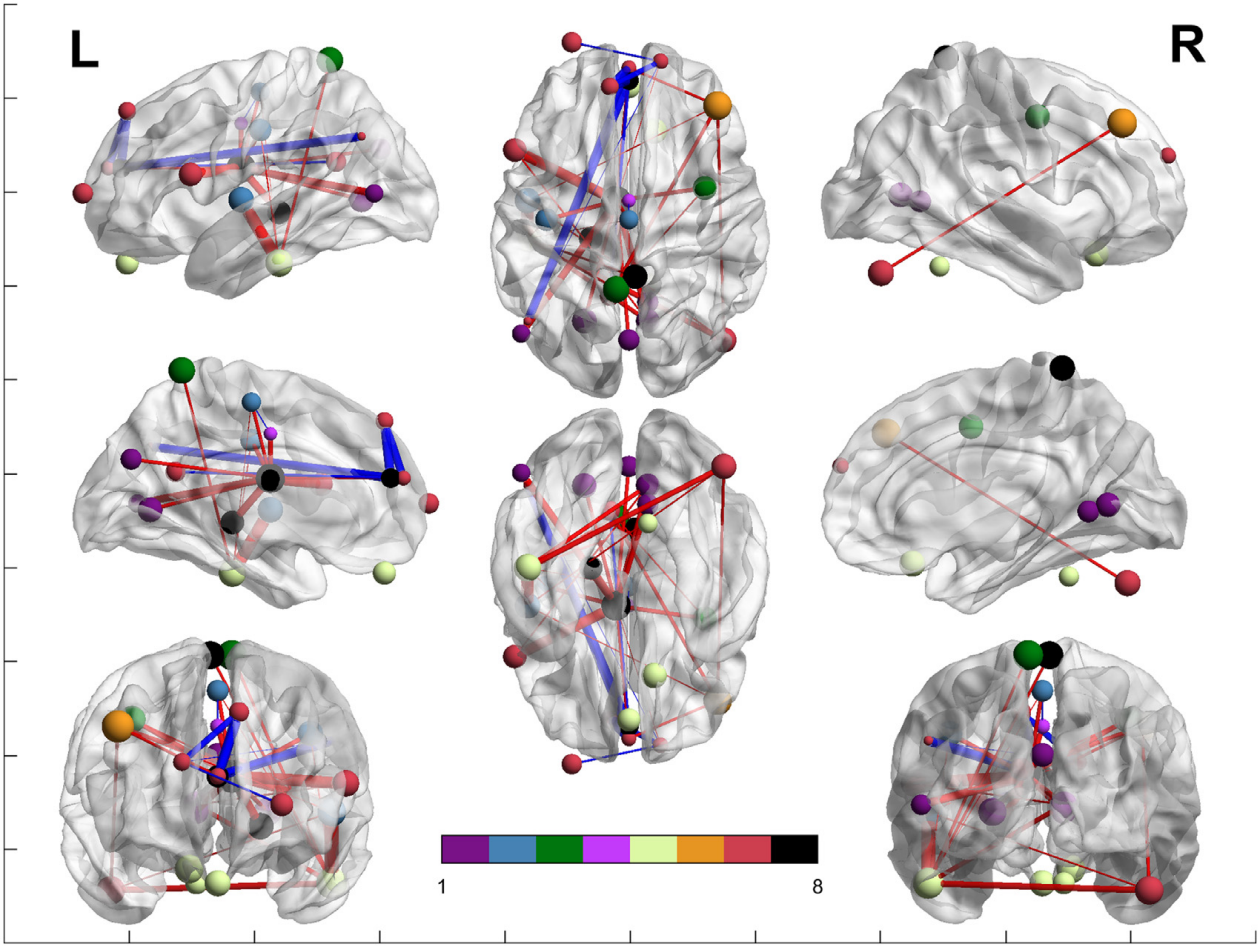
Network analysis between both groups. Colors represent the Yeo et al. (2011) networks, following their legend. Purple represents the ROIs included in the VN; blue represents the ROIs included in the SMN; green represents the ROIs included in the DAN; light purple represents the ROIs included in the VAN; yellow represents the ROIs included in the LN, orange represents the ROIs included in the FPN; red represents the ROIs included in the DMN; and black represents the ROIs not belonging to Yeo's network. Blue lines show areas that have significantly increased connectivity in control participants compared to DS. Red lines show areas that have increased connectivity in DS patients compared with controls. The brain networks were visualized with BrainNet Viewer (Xia et al., 2013).

Figure 2 shows the edge plot matrix, Figure 3 shows the spatial connectogram, and Figure 4 shows the brain network representation.

Regarding the nodes of other regions, the left thalamus shows widespread increased FC in the DS group: with all the nodes included in the VN, with some nodes of the SMN, DAN, VAN, DMN and the left hippocampus, which is not included in the Yeo parcellations. The right precuneus demonstrates also increased FC in the DS group with a node that conforms the LN, whereas the left hippocampus, as mentioned above, also shows increased FC with the left thalamus. Finally, the left cingulum anterior shows decreased FC in the DS group with areas of the DMN.

Regarding the VN, all the ROIs seem to be increased in the DS group. The first right lingual (shown in Figure 2) shows increased FC in the DS group with a region of the LN, a region of the DMN and with a region which is not included in the Yeo parcellations. The rest of the areas included in the VN show increased FC in the DS group with the left thalamus.

Regarding the SMN, the FC of the left superior temporal appears strongly increased with an area of the LN, an area of the FPN and an area of the DMN. The left Postcentral gyrus shows increased connections in the DS with an area of the LN and a ROI that do not pertain to the Yeo parcellations. Finally, the left supplementary motor area appears increased in the control group with the VAN's ROI whereas increased in the DS group with a ROI that pertains to the LN.

Concerning the DAN, all the connections seem increased in the DS group: the left precuneus with a region of the LN, and the right precentral gyrus with a region that do not pertain to the Yeo parcellations.

In relation to the VAN, the left cingulum mid appears to be decreased in DS with a region that pertains to the SMN, and decreased with the left thalamus.

Regarding the LN, only the temporal inferior left seems strongly increased in the DS group, with a region of the VN, with all the regions of the SMN, with a region of the DAN and DMN and finally with the right precuneus.

Referring to the FPN, the connections appear to be increased in the DS group. The right frontal mid show increased FC in the DS group with the SMN and with some regions of the DMN.

Finally, regarding the DMN, the left superior medial appears to be increased with the region of the FPN and with a region that does not pertain to the Yeo parcellations (left thalamus). However, shows decreased FC in the DS group with some regions of the DMN and with the left cingulum anterior. The left middle frontal gyrus appears increased with a region of the DMN. The cerebellum Crus 1 right appears increased in the DS group with a region of the SMN, a region of the LN and a region of the FPN. The inferior frontal gyrus opercular part appears increased in the DS group with a region of the VN and a region that is not included in the Yeo parcellations (left thalamus). The left precuneus appears increased in the DS group with a region of the DAN and the region of the FPN, whereas decreased with a region of the DMN and with the cingulum anterior left. The left angular only appears increased in the control group, in regions of the DMN and also with the cingulum anterior left. The left superior medial gyrus appears also increased in the control group with some regions of the DMN and also with the cingulum anterior left. Finally, the right precuneus appears increased in the DS group with the left thalamus (a region not included in the Yeo parcellations) whereas decreased with some regions of the DMN.

## Discussion

To our knowledge, this is one of the few studies on brain networks to investigate the FC of resting-state *f*MRI in DS. The present paper aimed to disentangle brain FC differences between DS and control participants.

Generally, the study showed the following results. (a) Alterations in DC were found. Increased DC was found in the temporal lobe, right frontal lobe, left caudate and left rectus. Decreased DC in the DS was found in the left frontal lobe. (b) Seed-based analysis revealed significant differences in many clusters. (c) Brain network analyses showed significant group differences between different areas of the Yeo et al. (2011) networks.

More concretely, in the first stage, DS participants showed increased DC in the temporal lobe, right frontal lobe, left caudate and left rectus. However, they showed decreased DC compared with controls in the right frontal lobe.

Decreased frontal lobe volume has been demonstrated in older and young adults with DS (Teipel et al., 2004; Powell et al., 2014). Moreover, structural abnormalities have been also reported in structures involving the thalamus and the caudate, regions that appear to be increased in DC in the DS group (McCann et al., 2021). It seems that decreased volume in this population can lead to FC abnormalities. In other populations, a link between structural and functional abnormalities has been demonstrated (Rogers et al., 2018; Valenti et al., 2020). Moreover, the structures that display abnormal DC are engaged in functions that are altered in DS, as language and executive functions (Hamburg et al., 2019).

It is also important to compare our results with those of Pujol et al. (2015), who also studied DC. In this sense, they found increased DC in the DS in the ventral anterior cingulate cortex (ACC) and decreased DC in the dorsal ACC. Both regions were also increased in DS (cluster of the left caudate) and decreased (cluster of the left frontal lobe) in our study. Moreover, the decreased DC in the medial frontal cluster reported by Pujol et al. (2015) was also found in our study (cluster left frontal lobe). Pujol et al. (2015) reported higher DC in DS in the right amygdala, which also is congruent with our study (cluster left caudate) and decreased DC in DS in areas of the posterior insula.

Regarding seed-based FC analyses, hyper connectivity in DS was found in most of the seeds used, finding high congruence with the results of DC analyses. It is important to highlight the importance that may have the left thalamus in the neuropathology of DS, because of the hyper connectivity found with other structures of the brain. This structure is implied in executive and memory functions (Stagni et al., 2020), which are impaired in DS, and it seems that is particularly sensitive to effects of aging, finding a loss of neurons and volume (Perry et al., 2019) in older persons with DS, as well as other abnormalities related to brain amyloid (Keator et al., 2020). These structural alterations could be related to the hyperconnectivity found regarding this structure with other areas. It is important also to highlight the big presence of subcortical structures in the significant differences between both populations as the thalamus and the caudate, which are especially important for the cortico-striatal-thalamo-cortical (CSTC) circuits. These regions have been linked with important neuropsychological deficits and neurodevelopmental diseases such as Autism Spectrum Disorder or Attention deficit/Hyperactivity Disorder (Riva et al., 2018).

Significant differences were found between DS and controls regarding brain network analyses. Within network connectivity was only found to be altered in the DMN, but significant differences were found between all the networks regarding between network connectivity.

More concretely, regarding the VN, hyperconnectivity was found in the links between the VN and some regions of the DMN and LN, as well as with the left thalamus. When we used the SMN as a seed, the brain networks also seemed altered in DS; concretely, they were increased with the LN, FPN, DMN and the left thalamus, whereas decreased in the VAN. The DAN also seems disrupted in DS, finding increased between network FC: The connections of the DAN with the LN, DMN and the left thalamus seemed to be increased in DS. When using the VAN as a seed, those networks linked with the left thalamus appeared to be increased in the DS while the networks linked with the SMN appeared to be decreased. Concerning the LN, only increased connections were found in DS involving areas of the VN, SMN, DAN, DMN and other regions. Regarding the FPN, links with regions of the SMN and DMN are increased in DS

Finally, concerning the DMN, on the topic of between network connectivity, networks that link with the DAN, SMN, VAN, VN, LN and FPN seem to be increased in DS, as well as the left thalamus- Interestingly, significant differences in within network connectivity were found in this network, only decreased in DS. Finally, the FC between the DMN and the structure of the left anterior cingulum was also decreased in DS.

Vega et al. (2015) also found results congruent with those presented in this study, despite their study including whole-brain Yeo et al. (2011) networks and with a small sample (*n* = 10). In this sense, they also found increased FC between the SMN and the DMN as well as increased FC between the SMN and the FPN. Moreover, they found increased FC between the LN and the DAN. However, Vega et al. (2015) found increased FC between the SMN and FPN, as well as between the DAN and FPN, whereas in our study these results were not reported. These different results could be explained because in our study there is only one ROI included that pertains to the FPN, which shows increased FC with areas of the SMN and DMN, but in the study of Vega et al. (2015) the whole network is included. The FPN could be underrepresented in our study and this fact could explain the differences between our study and the one published by Vega et al. (2015). Finally, regarding the three hyperconnected links of the FPN in DS demonstrated in our study, could be consistent with the results of the Arizona Cognitive Test Battery (Edgin et al., 2010), showing cognitive deficits in the frontal lobe functions, as pointed by Vega et al. (2015).

Interestingly, it is important to highlight the disrupted FC pattern in the DMN found in the DS group. Intrinsic FC in the DMN is certainly altered, finding a clear pattern of hypoconnectivity in the DS group within areas of the anterior DMN (including frontal medial areas). Rosas et al. (2021) also studied the intrinsic FC of the DMN in this population, and found abnormalities also congruent with our study, finding an anterior-posterior dissociation in the DMN: the anterior parts of the DMN (in our study, the left frontal superior medial gyrus and the left frontal middle gyrus) were disconnected in the DS group from the posterior parts of the brain (precuneus and left angular gyrus). Koenig et al. (2021) also found decreased FC in the majority of connections between the anterior and posterior aspects of the cingulate cortex. These results could be linked to the structural abnormalities found in this population in the cerebellum (Guidi et al., 2011; Lee et al., 2020). Contrarily, between network connectivity of the DMN with the other networks seems increased in the DS group, finding a clear pattern of hyper connectivity with all of the other Yeo et al. (2012) networks (VN, SMN, DAN, LN and FPN). This pattern was congruent with Wilson et al (2019), who found increased FC in the DS group from the DMN to the rest of the brain.

At this point, it is important to mention a recently published study by Csumitta et al. (2022) also with young DS using resting-state *f*MRI. They found significant differences in 18 ROIs, and they classified them also using Yeo et al. (2011) parcellations. Left inferior temporal, right fusiform, left middle frontal and left inferior frontal, among others, were also found to be significant in our study. Moreover, their regions also included cerebellar ROIs, which also were significantly different among controls and DS. However, they found in all the areas increased FC in DS, whereas our findings are depending on the area, congruent with other studies.

Findings in this study confirm some of the results of already published papers, innovating in some points. However, there are some limitations that are worth mentioning, such as the sample used in this study, which is similar to other neuroimaging studies but still is poor. Two subjects were discarded for movement, which is also typical in this population, and movement, despite being well controlled, could hinder the results. Therefore, more studies in this line should be published to confirm the results.

Despite the limitations, to our knowledge, this is the first study of brain networks in DS. Some studies have used seeds to study FC, but no one has used brain networks. Despite using a small sample, the corrections applied in this analysis for multiple comparisons are stringent, and the effect sizes of all the comparisons are large. Moreover, the results are in line with those of another study performed in this field.

## Conclusions

The results of this study demonstrate DC alterations in frontal and temporal areas that could be related with the neuropsychological profile of DS (with major dysfunction in executive functions and language). Moreover, the results that show abnormalities in DC and seed-based FC analyses are related with structural abnormalities already proven in this population. More studies linking structural and functional brain abnormalities are needed to demonstrate this association in this population. Regarding brain networks analyses, results show a disrupted pattern of between network connectivity involving all the Yeo networks, but only in the DMN there is altered within-network FC, finding hypoconnectivity in many areas of the DMN. Regarding the results in within network connectivity of the DMN, we confirm an anterior-posterior dissociation in this network already described in other studies. This means that connectivity in DS is altered, and these abnormalities could be related to the characteristics of the disease (low ID, physiological features, decreased volume of different areas of the brain such as the cerebellum, etc.). It is important to mention that most differences found in connectivity between DS and controls are located within the Yeo parcellations. Moreover, regions that seem more disconnected should be targeted to plan therapeutic interventions to promote increased connections. The three methods used in this study (DC, seed-based FC and brain network analysis) have proven to be useful tools to disentangle brain abnormalities in this population.

## Data availability statement

The data that support the findings of this study are available on request from the corresponding author. The data are not publicly available due to privacy or ethical restrictions.

## Funding statement

This study was funded by the Spanish Ministry of Science, Innovation and Universities (PGC2018-095829-B-I00).

## Ethics approval statement

The protocol was approved by the Bioethical Committee of the Universitat de Barcelona (03/16/2017).

## Patient consent statement

Informed consent was also acquired from all DS and control participants, as well as from the guardians in legal charge of every person with DS.

## CRediT authorship contribution statement

**Cristina Cañete-Massé:** Conceptualization, Data curation, Methodology, Investigation. **Maria Carbó-Carreté:** Conceptualization, Data curation, Supervision. **Maribel Peró-Cebollero:** Conceptualization, Supervision. **Shi-Xian Cui:** Conceptualization, Supervision. **Chao-Gan Yan:** Conceptualization, Methodology, Investigation. **Joan Guàrdia-Olmos:** Conceptualization, Resources, Methodology, Investigation.

## Declaration of Competing Interest

The authors declare that they have no conflicts of interest.
